# Preferred music listening is associated with perceptual learning enhancement at the expense of self-focused attention

**DOI:** 10.3758/s13423-022-02127-8

**Published:** 2022-06-06

**Authors:** Pietro Sarasso, Paolo Barbieri, Elena Del Fante, Ludovico Bechis, Marco Neppi-Modona, Katiuscia Sacco, Irene Ronga

**Affiliations:** BIP (BraIn Plasticity and behaviour changes) Research Group, Department of Psychology, University of Turin, Turin, Italy

**Keywords:** Neuroaesthetics, EEG, ECG, Attention, MMN, Learning, Heartbeat, Music

## Abstract

Can preferred music listening improve following attentional and learning performances? Here we suggest that this may be the case. In Experiment 1, following preferred and non-preferred musical-piece listening, we recorded electrophysiological responses to an auditory roving-paradigm. We computed the mismatch negativity (MMN – the difference between responses to novel and repeated stimulation), as an index of perceptual learning, and we measured the correlation between trial-by-trial EEG responses and the fluctuations in Bayesian Surprise, as a quantification of the neural attunement with stimulus informational value. Furthermore, during music listening, we recorded oscillatory cortical activity. MMN and trial-by-trial correlation with Bayesian surprise were significantly larger after subjectively preferred versus non-preferred music, indicating the enhancement of perceptual learning. The analysis on oscillatory activity during music listening showed a selective alpha power increased in response to preferred music, an effect often related to cognitive enhancements. In Experiment 2, we explored whether this learning improvement was realized at the expense of self-focused attention. Therefore, after preferred versus non-preferred music listening, we collected Heart-Beat Detection (HBD) accuracy, as a measure of the attentional focus toward the self. HBD was significantly lowered following preferred music listening. Overall, our results suggest the presence of a specific neural mechanism that, in response to aesthetically pleasing stimuli, and through the modulation of alpha oscillatory activity, redirects neural resources away from the self and toward the environment. This attentional up-weighting of external stimuli might be fruitfully exploited in a wide area of human learning activities, including education, neurorehabilitation and therapy.

**Supplementary Information:**

The online version contains supplementary material available at 10.3758/s13423-022-02127-8.

## Introduction

In a 1993 *Nature* paper, Rauscher et al. (1993) showed that listening to Mozart's sonata K448 enhanced spatiotemporal reasoning. Despite some conflicting results, subsequent studies substantially confirmed the presence of the so-called Mozart effect (Jenkins, 2001). It has been suggested that cognitive enhancements following music listening might be mediated by the arousal/attentional state of participants following aesthetic appreciation of music, rather than being caused by music per se (Thompson et al., 2001). Indeed, beauty perception may be associated with a specific attentional attitude (i.e., the “aesthetic attitude”) focused on the perceptual activity for its own sake (Menninghaus et al., 2019), which diverges from everyday pragmatic, interest-driven perception (Kemp, 1999; Nanay, 2016; Pearce et al., 2016). Accordingly, aesthetic appreciation has been linked to attention enhancement and knowledge acquisition (Schoeller & Perlovsky, 2016; Van de Cruys & Wagemans, 2011). Previous studies demonstrated that behavioural and electrophysiological markers of attention and perceptual learning, such as the mismatch negativity (MMN) response, are enhanced in response to more appreciated visual (Sarasso et al., 2020b) and auditory stimuli (Sarasso et al., 2019, 2021a, 2021b). However, whether or not this positive effect on perceptual learning can be protracted even after an aesthetic experience has not been systematically investigated yet. The present behavioural-EEG study aimed to: (1) test whether perceptual learning of sensory regularities is enhanced following preferred music listening, and (2) investigate the role of attentional deployment (toward the self vs. the environment) in the genesis of cognitive enhancement following music listening.

To this purpose, we designed two experiments, an EEG study (Experiment 1) employing a well-validated roving paradigm (see Methods) to measure the ability of the sensory system to learn statistical regularities of the sensory environment (Garrido et al., 2016; Ostwald et al., 2012), and a behavioural study (Experiment 2) exploring participants’ interoceptive awareness (Ainley et al., 2012) following music listening. To modulate participants’ aesthetic attitude in the present experiments, we employed four classical music pieces played forwards and backwards. We collected subjective aesthetic ratings for each of the eight musical pieces and we compared results obtained after listening to more and less appreciated music, grouped according to subjective ratings (Sarasso et al., 2021b).

In Experiment 1, following music listening, participants attended to a stream of sounds varying in their frequency (Hz), while we recorded their EEG. We then computed the MMN, a differential wave obtained by subtracting the event-related response to standard (i.e., repeated) sounds from that of deviant sounds (Näätänen et al., 2007). The MMN reflects activations in the posterior auditory cortex and the inferior frontal gyrus, and peaks around 100–250 ms post deviancy onset (Molholm et al., 2005). The MMN is commonly considered as a neurophysiological signature of perceptual learning, indicating the update of the predictive models generated by the nervous system to anticipate the sensory regularities occurring in the environment (Lieder et al., 2013). To further investigate the possible modulation of the neural encoding of sensory regularities/surprise induced by preferred music listening, we performed a point-by-point correlation (Novembre et al., 2018; Sarasso et al., 2021b) between EEG single-trial amplitude fluctuations and a theoretic index of perceptual learning, i.e., the Bayesian Surprise (Baldi & Itti, 2010). The Bayesian Surprise describes the potential learning occurring when the brain has “assimilated” the new input to update its predictive model of the environment (Faraji et al., 2018). It is computed as the divergence between prior and posterior beliefs following each sound and can be conceived as the quantity of information conveyed by single stimuli, assuming that the perceiver is an ideal Bayesian observer (see Methods and Online Supplemental Material (OSM)). Thereby, the correlation between brain responses and Bayesian Surprise captures how much the cognitive system is “attuned” to sensory information.

Altogether, MMN amplitude fluctuations and the single-trial correlation with Bayesian Surprise index represent validated methods to track participants’ learning abilities throughout the experiment. Thanks to these biomarkers, we will be able to measure the possible outcomes of the aesthetic attitude, but we will not uncover the neural mechanisms establishing such an attitude. To this aim, we recorded participants’ neural activity during music listening. Previous studies showed increased alpha oscillatory activity immediately after music listening (Jaušovec et al., 2006; Verrusio et al., 2015). Crucially, alpha power at rest is a powerful predictor of participants’ attentional state and ability to learn (Jann et al., 2010; Sigala et al., 2014). It has been demonstrated that approximately 40% of the inter-subject variability in perceptual learning can be explained by the resting-state posterior parietal alpha intrinsic oscillations (Freyer et al., 2013). Therefore, to verify whether listening to preferred music was able to enhance alpha oscillatory activity, we explored resting-state EEG spectral profiles recorded during the last minutes of musical pieces’ attendance.

We previously suggested (Sarasso et al., 2020a) that the beauty-induced aesthetic attitude toward learning might be paralleled by a transient disinterest in self-related utilitarian concerns (for a discussion on disinterestedness in neuroaesthetics, see Vassiliou, 2020). To test this hypothesis, in Experiment 2, we investigated whether the possible attentional modulations and perceptual learning enhancement following preferred music listening were associated with changes in self-focused attention. Importantly, interoceptive accuracy in hearth beat detection (HBD) appeared increased by the attentional focus on the self (Pollatos et al., 2016) and is commonly employed to assess attentional focus toward the self versus the environment (García-Cordero et al., 2017). Furthermore, it has been demonstrated that priming stimuli that direct attention toward the self (Ainley & Tsakiris, 2013; Ainley et al., 2012; Maister & Tsakiris, 2014; Weisz et al., 1988), or even pathological self-concern in panic/anxiety disorders (for a recent review, see Brewer et al., 2021), can enhance the accuracy in HBD tasks. Therefore, to test whether the beauty-induced aesthetic attitude may affect self-focus attention, we designed a HBD task performed by participants listening to the same musical stimuli employed in Experiment 1.

If the appreciation of preferred musical stimuli is able to induce an aesthetic attitude following music listening, we should observe an enhancement of participants’ attention and perceptual learning abilities in Experiment 1. More specifically, we expect: (1) MMN to be larger after listening to preferred versus non-preferred music in the roving paradigm; (2) the correlation between EEG responses single-trial amplitudes and Bayesian Surprise to be stronger after listening to preferred versus non-preferred music, thus indicating a better neural encoding of sensory information (Experiment 1). If an aesthetic attitude is established during preferred music listening, by recording participants’ neural activity, we should pinpoint the neural mechanism responsible for such an attitude. We predict to observe (3) a resting-state alpha power to be larger during listening to preferred music (Experiment 1). Importantly, the enhancement of participants’ attention toward the sensory environment, resulting in an amplified perceptual learning, may be paralleled by the detriment of self-focused attention, thus decreasing interoceptive awareness in the HBD task (Experiment 2). Therefore, we predict (4) the accuracy measured by the HBD task to be lower after listening to preferred music (Experiment 2).

## Experiment 1 (EEG experiment)

### Methods

#### Participants

Eighteen right-handed healthy subjects participated in Experiment1 (11 women, mean age: 25.833 years; SD: ± 1.790; mean years of education: 18.833; SD: ± 1.505). All participants gave their written informed consent to participate in the study, which conformed to the standards required by the Declaration of Helsinki and was approved by the Ethics Committee of the University of Turin (Prot. n. 121724 – 01/03/18). Participants were not compensated for taking part in the experiment.

Sample size (N = 18) was a priori determined to match the average number of participants involved in previous studies highlighting evoked-response potential (ERP) modulations driven by aesthetic appreciation (Sarasso et al., 2019, N = 22; Sarasso et al., 2020b, N = 13; Sarasso et al. 2021b, N = 18; average = 17.7).

#### Stimuli and experimental design

##### Musical stimuli

The eight musical stimuli we employed were composed of four classical music excerpts cropped to a 5-min length, and the same four excerpts played backwards. We selected the following classical music pieces (see OSM): Debussy’s Violin Sonata in G Minor Mvt.1 (allegro vivo); Chopin’s Nocturne in B-Flat Minor Op.9 N.1 (larghetto); Mozart’s Piano Sonata No.11 in A Major K.331 (andante grazioso); Ravel Piano Concerto in G major (*allegramente*). These musical pieces were chosen for their low familiarity for a non-expert public. Indeed, none of our participants could recognize or remember them. As assessed by previous studies (Fritz et al., 2013), reversed music is effective in modulating participants’ aesthetic appreciation while maintaining similar acoustic features (e.g., rhythm and pitch). Moreover, other than direction (i.e., forward or backward), musical stimuli differed by two additional variables: key (i.e., Major or Minor) and tempo (i.e., fast or slow). Musical pieces were paired for key and tempo (two with major and two with minor key; two with fast and two with slow tempo). Stimuli were matched for key, tempo and the direction of presentation to control for whether aesthetic preferences were actually driving the observed effects, or whether instead other objective features of the music stimuli were the responsible for the results. As reported in the *Data analyses* section, we compared ACC and aesthetic judgements (AJs) following forward versus backward, major versus minor and fast versus slow music listening. Furthermore, to avoid any sequence effect due to specific orders of presentation, musical pieces were played in a random order across the eight blocks. Therefore, the sequence of presentation of the eight musical excerpts was different for each subject.

##### Procedures

The experiment was based on a within-subject design and consisted of eight blocks of two subsequent tasks. First, for each block, participants passively listened to 5 min musical excerpts (i.e., ‘Musical Listening’; subjects passively listened to 40 min of music or reversed music in total in the whole experiment). Subsequently, subjects were asked to express an aesthetic judgment using a Likert scale ranging from 1 to 9 (where 1 corresponded to “The ugliest music I can imagine” and 9 corresponded to “The most beautiful music I can imagine”; Sarasso et al., 2019, 2020b, 2021) by pressing the corresponding key on the computer keyboard. AJs were registered for each block/musical piece (E-Prime 2.0 software, Psychology Software Tools, Inc. USA). After each musical excerpt, subjects listened to a stream of sounds presented according to a roving paradigm while we registered their EEG (see *EEG Mismatch Negativity Task*). Furthermore, we recorded the resting-state electrophysiological activity during the last 2 min of passive forwards or backwards music listening. Resting state EEG data served as input for the analyses in the frequency domain (see Data analysis). During the whole experiment, participants sat at a table with eyes open, in front of a 53-cm (diagonal) computer screen. The screen centre was aligned with the subjects’ trunk midline. Participants’ arms were resting on the ipsilateral leg during the MMN roving paradigm.

##### EEG Mismatch Negativity Task (MMN)

The EEG Mismatch Negativity Task is based on a roving auditory paradigm (Baldeweg et al., 2004; Ostwald et al., 2012), with standard and deviant sounds differing in their frequency (Hz). The roving paradigm was designed to investigate the MMN differential wave, a consolidated neurophysiological marker of implicit perceptual learning of sensory regularities (Garrido et al., 2016; Lieder et al., 2013). MMN responses were registered while subjects listened to sounds created with Csound (https://csound.com/). The software allowed us to select the frequency of the synthetic sounds composing the roving sequence. Low-pitch and high-pitch sounds had a frequency of 600 and 1,200 Hz, respectively. Sound sequences were presented by Eprime V2.0 presentation software (Psychology Software Tools Inc., Pittsburgh, PA, USA). Each sound was played for 50 ms via loudspeakers. Loudness of sounds was set at a comfortable level (≅ 70 dB) and was kept equal across subjects and experiments.

**Fig. 1:**
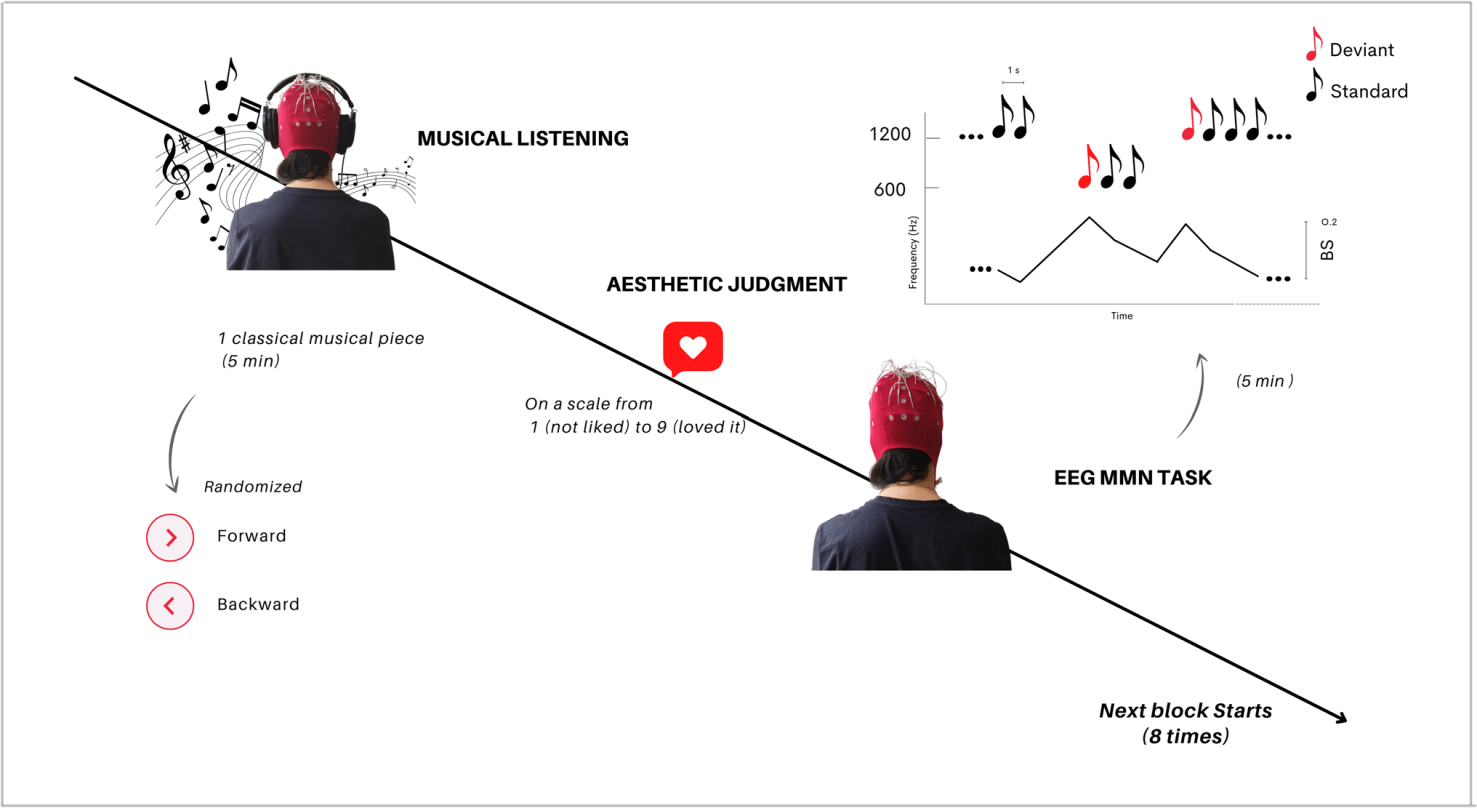
Experiment 1 – experimental paradigm. The sequence of deviant and standard sounds presented according to a roving paradigm followed 5 min of music (or reversed music) listening. Right after the end of each musical piece we collected aesthetic ratings. The whole procedure was identically repeated eight times, once for each musical stimulus. The EEG was registered during the whole experiment

Sound sequences consisted of trains of 288 sounds (duration 50 ms) per run, each lasting 302.4 s (sounds were played at a 1-Hz frequency – block whole duration 288 s + 50 ms duration of each sound). While listening to the sound sequences, participants were asked to remain silent and look straight ahead at a central fixation cross on the computer screen. Differently from traditional oddball paradigms, in roving protocols each stimulus type has the same probability to occur, thus allowing dissociation of genuine effects of Bayesian perceptual learning from rarity-driven modulations. In roving paradigms, different stimuli (high-pitch and low-pitch tones in our case) can represent both Deviant and Standard stimuli (Fig. 1), as opposed to traditional oddball paradigms (Näätänen et al., 1978) where the repeated presentation of standard sounds is occasionally interrupted by the occurrence of physically different deviant sounds. In our case, high-pitch and low-pitch intervals were presented in consecutive trains of alternating pitch with a constant inter-stimulus interval of 1 s (in accordance with previous studies employing similar inter-trial intervals; Ostwald et al., 2012). Any time a change in the stimulation stream occurs (i.e., the transition from a high-pitch to a low-pitch stimulus train or vice versa), the first stimulus of the new train constitutes a Deviant event, since it differs in frequency (Hz) from the preceding train of stimuli, which are therefore considered Standard. The length of the trains of high-pitch and low-pitch intervals was chosen according to a pseudo-random order, so that both the number of presentations and the average value of the Bayesian surprise (see *Bayesian perceptual surprise computation*) were equal across pitch types (i.e., high or low; Fig. 1). Moreover, the ratio between Standard (80%) and Deviant (20%) trials was kept equal across blocks. The average length of consecutive equal sounds was 5.143 ± 3.739, with a maximum of 16 equal consecutive sounds and a minimum of one sound.

##### Bayesian perceptual surprise computation

For a detailed description of the mathematical computations please refer to the OSM. Similar to previous studies (Baldi & Itti, 2010; Ostwald et al., 2012), to relate single-trial EEG signals to Bayesian perceptual learning, we computed Bayesian Surprise for each single trial using a sequential Bayesian learning algorithm of stimulus probabilities, thus obtaining 288 (i.e., the total number of sounds composing the sequence presented in each session of the EEG MMN task) estimated surprise values. The model assumes that the brain implements a trial-by-trial Bayesian parameter learning scheme starting from an uninformative prior and computes Bayesian Surprise as the divergence between the parameter prior and posterior probability density functions at the single-trial level. Following Ostwald et al. (2012), we use a variant of this model that assumes an exponential forgetting of stimuli that are observed in the distant past (we set the forgetting parameter to τ = 4, which was shown to best describe neural activity; Ostwald et al., 2012). The degree of perceptual learning at the n-th trial is then defined as Bayesian Surprise, i.e., the Kullback–Leibler divergence between the prior and posterior distribution over the probability of observing a high-pitch interval on the n-th trial (Cover & Thomas, 1991). Surprise values are larger when the Deviant stimulus (e.g., a high-pitch sound) comes after longer trains of identical stimuli (e.g., low-pitch sounds), since strong evidence for Standard sounds (e.g., low pitch) accumulates with increased precision and the divergence between prior and posterior probability distributions results is larger.

#### Data analysis

##### Aesthetic judgements

We first analysed behavioural results from the aesthetic judgment task. This allowed us to identify the preferred musical pieces for each subject. We divided the eight musical pieces into two categories (i.e., preferred and non-preferred), for each participant separately based on her/his ratings. The group-level effect of preference was evaluated by comparing the average ratings of the four preferred and non-preferred musical pieces. Thus, we performed a two-tailed t-test (N = 18) between the preferred and the non-preferred musical pieces. Moreover, to exclude that the results from subsequent analyses were influenced by musical stimulus objective features, rather than by subjective preferences, we compared average aesthetic judgements for forward versus backward music, major versus minor music, and fast versus slow tempo music. Therefore, three additional control two-tailed t-tests were performed to investigate whether key, tempo and direction significantly influenced aesthetic judgements.

##### Electrophysiological recordings and EEG preprocessing

EEG data were collected during the eight runs of the EEG MMN task and during the last 2 min of music listening, with 32 Ag-AgCl electrodes placed on the scalp according to the extended International 10–20 system and referenced to the nose. Electrode impedances were kept below 5 kΩ. The electro-oculogram (EOG) was recorded from two surface electrodes, one placed over the left lower eyelid and the other placed lateral to the outer canthus of the left eye. EEG activity was recorded with a HandyEGG amplifier (HandyEEG e SystemPlus Evolution, Micromed, Treviso – IT) and continuously digitized at a sampling rate of 1,024 Hz.

##### MMN analyses

Data collected during the EEG MMN Task were off-line pre-processed with Letswave6. Data were segmented into epochs of 1 s, including 200-ms pre-stimulus and 800-ms post-stimulus intervals. Epochs were band-pass filtered (0.5–40 Hz) using a fast Fourier transform filter (in accordance with previous literature exploring MMN; Ostwald et al. 2012). Filtered epoched data were baseline corrected using the interval from -0.15 to 0 s as reference. Ocular artefacts were eliminated using Independent Component Analysis (ICA; Jung et al., 2000). ERPs were divided according to the participants' subjective aesthetic appreciation as measured by the aesthetic evaluation following each musical stimulus, resulting in two conditions: preferred and non-preferred. That is, independent of their objective features (e.g., forward or backward presentation), the four most appreciated musical excerpts corresponded to the preferred condition, while the four least appreciated excerpts were assigned to the non-preferred condition. ERPs belonging to the same condition (i.e., preferred or non-preferred) and to the same deviance condition (i.e., Standard vs. Deviant) were then averaged, to obtain four average waveforms for each subject (i.e., Preferred Standard, Preferred Deviant, Non-preferred Standard, Non-preferred Deviant).

MMN responses were obtained by subtracting the ERPs elicited by standard intervals from those elicited by deviant intervals (Näätänen et al., 2007). Importantly, in this analysis we included only the last standard trial for each stimulus train occurring before deviant trials (N = 52 per run; Ostwald et al., 2012). In this way, in the MMN analysis, the number of standard and deviant trials was matched. Single participants’ MMN registered on single channels were entered in subsequent group-level analyses. We were interested in testing for possible differences in MMN registered after listening to more and less appreciated musical stimuli.

In the present study we employed point-by-point statistical tests. Point-by-point analyses represent a statistical approach common in EEG studies (Bruno et al., 2020; Harris et al., 2013; Novembre et al., 2018; Ronga et al., 2013), directed to highlight possible differences between conditions across the whole epoch time-course, without any a priori assumption. One statistical comparison for each time point composing a waveform is performed. To correct for multiple comparisons, cluster-based permutation testing approach (1,000 random permutations) was employed to each point-by-point analysis (Maris & Oostenveld, 2007). The thus obtained clusters of significance represent the result of the point-by-point analyses, corrected by permutation testing. For a similar statistical approach, please refer to Sarasso et al. (2019, 2020b).

To test for possible differences in MMN registered after listening to preferred versus non-preferred musical stimuli, we performed a point-by-point t-test (Novembre et al., 2018), with clustersize-based permutation correction for multiple comparisons based on temporal consecutivity and spatial adjacency (1,000 permutations; alpha level = 0.05; percentile of mean cluster sum = 95; minimum number of adjacent channels = 2), on differential MMN waves (Deviant-Standard). The test compared single subjects’ MMN amplitudes corresponding to the preferred and non-preferred conditions, for each channel separately. This allowed us to identify time-clusters containing brainwave amplitudes that significantly differed between preferred and non-preferred conditions.

##### Trial-by-trial correlation with Bayesian Surprise

Pre-processed epochs and Bayesian Surprise values corresponding to single trials constituted the input of a point-by-point trial-by-trial correlation analysis (Novembre et al., 2018; Sarasso et al., 2019, 2020b). For each participant and for each block separately, the analysis computed the correlation between Bayesian Surprise and trial-by-trial (N = 288) fluctuations of the EEG signal amplitude registered at single channels. The outcome of the correlation analysis was eight 1-s long (from 0.2 s pre-onset to 0.8 s post-onset) time series of r-values for each channel and for each subject. R-values were then averaged across blocks according to subjective preference, resulting in two time series, one for preferred and one for non-preferred music. This constituted the input for a group-level two-tailed point-by-point t-test with permutation-based correction for multiple comparisons (1,000 permutations; alpha level = 0.05; percentile of mean cluster sum = 95). The test compared correlation coefficients corresponding to the preferred and the non-preferred conditions. Possible differences in r-values between amplitudes and Bayesian Surprise would indicate a different encoding of sensory surprise during the perception of sound sequences in the two different preference conditions.

##### Analyses in the frequency domain

EEG recordings collected during passive music listening served as input to analyse possible differences following preferred and non-preferred music in the frequency domain and to explore whether such difference might be predicted by a specific frequency modulation observed in the last two minutes of music listening. The off-line pre-processing and analyses were conducted with Letswave6 toolbox (Nocions, Ucl. BE) for Matlab (Mathworks, Inc., USA). The EEG data corresponding to each of the eight musical excerpts were divided into 20 segments of 4 s registered at the end of each excerpt (i.e., 80 s at the end of each musical piece; Verrusio et al., 2015). Each segment (i.e., 40 segments) was band-pass filtered (0.5–70 Hz) and notch filtered (50 Hz) using a Fast Fourier transform filter. Filtered epoched data were transformed in the frequency domain using a Fast Fourier Transformation (FFT). Epochs served as input for calculating the power in the various EEG frequency bands from 1 to 50 Hz (delta = 1–3.5 Hz; theta = 4–7.5 Hz; alpha = 8–12.5 Hz; low beta = 13–19.5 Hz; high beta = 20–29.5 Hz; and gamma = 30–50 Hz). We then averaged the 20 FFT transformed segments (each corresponding to a frequency power spectrum) obtaining an average spectrum for each block/musical excerpt. The average spectral profiles were then averaged according to the participants' subjective aesthetic appreciation, resulting in two conditions: preferred and non-preferred. As previously described in the MMN analyses, independently from their objective features (e.g., forward or backward presentation), the four most appreciated musical excerpts corresponded to the preferred condition, while the four least appreciated excerpts were assigned to the non-preferred scenario. The four average power spectrums corresponding to single blocks/musical stimuli that belonged to the same condition (i.e., preferred or non-preferred) were then averaged, to obtain two average spectral profiles for each single participant (i.e., preferred and non-preferred).

**Fig. 2:**
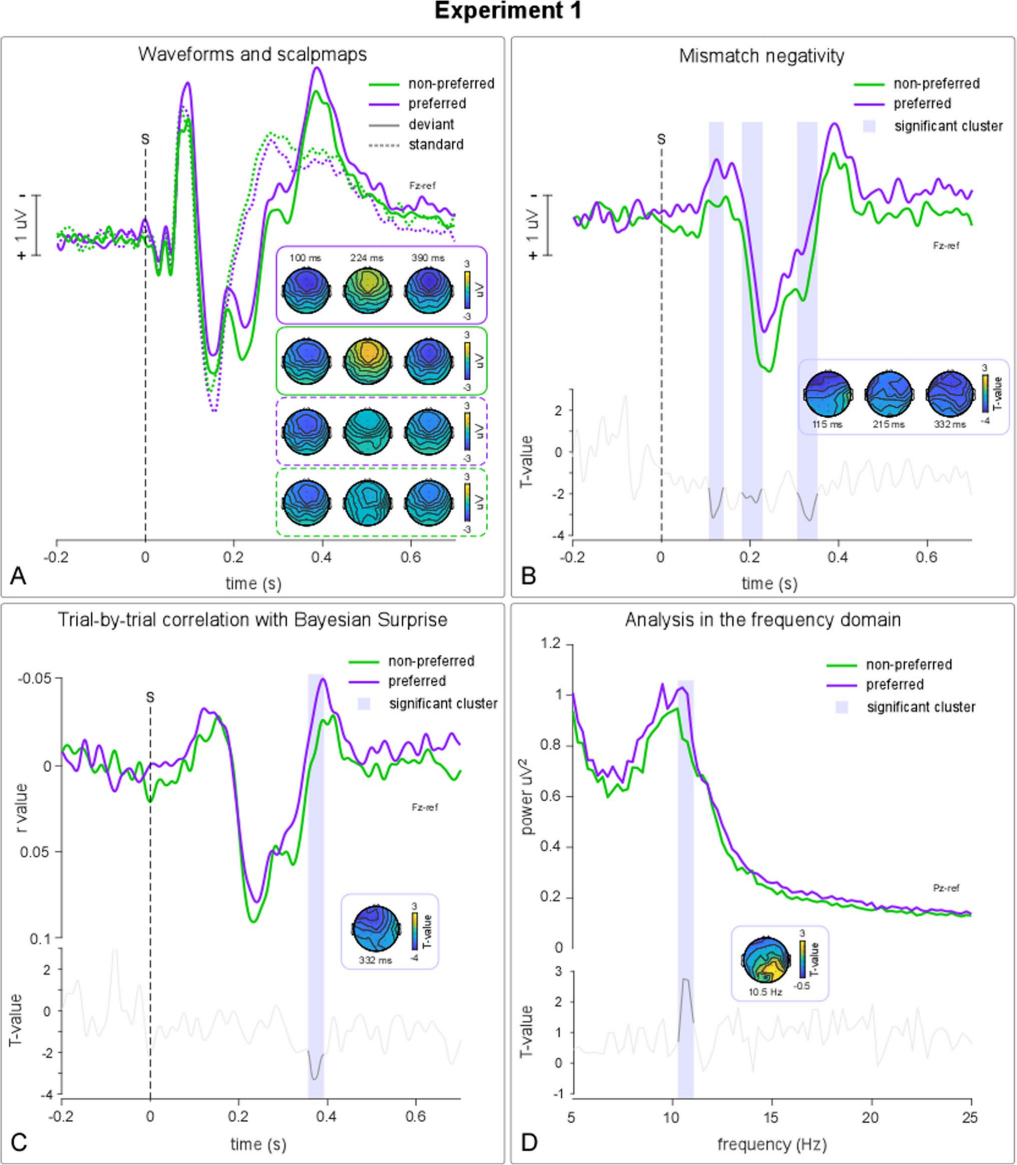
Experiment 1 – results. In panel **A**, grand average (N = 18) responses triggered by standard and deviant sounds after listening to preferred and non-preferred music are depicted. Scalpmaps represent average amplitudes across deviancy and preference conditions at 100, 224 and 390 ms post-onset, corresponding to the N1, P2 and N4 peak latencies. MMN differential waveforms (deviant-standard) are represented in panel **B** and peaked at around 150 ms post-onset. T-values from the point-by-point t-test comparing MMN after preferred and non-preferred music listening are shown at the bottom of the panel. Significant t-values are highlighted in black and grey shaded areas and correspond to significant clusters surviving cluster-based permutation correction. Scalpmaps represent t-values within significant clusters. Panel **C** shows average r-values between amplitudes at Fz and Bayesian Surprise and t-values from the point-by-point trial-by-trial correlation analysis. Shaded areas correspond to significant clusters surviving cluster-based permutation correction as revealed by the pint-by-point t-test comparing average r-values corresponding to preferred vs. non-preferred music listening. The scalpmap represents t-values within the significant cluster. Panel **D** shows the resting state EEG spectral profile registered during the last 80s of music listening. Shaded areas correspond to significant clusters surviving cluster-based permutation correction as revealed by the pint-by-point t-test comparing the spectrum corresponding to preferred vs. non-preferred music listening. The scalpmap represents t-values within the cluster centred over the upper alpha frequency (10.5 Hz)

Single participants’ average power spectrum registered on single channels were entered in subsequent group-level analyses. We performed a point-by-point t-test (Novembre et al., 2018), with clustersize-based permutation correction for multiple comparisons based on temporal consecutivity and spatial adjacency (1,000 permutations; alpha level = 0.05; percentile of mean cluster sum = 95; minimum number of adjacent channels = 2), on average spectrums (preferred vs. non-preferred). The test compared single subjects’ frequency spectral power corresponding to preferred and non-preferred conditions at each time point, for each channel separately. This allowed us to compare the power spectral profiles registered after listening to the most and the least appreciated musical pieces, in all the frequency bands of interest. Lastly, in order to obtain a visually intuitive representation of the effect of aesthetic appreciation on EEG spectral profiles, single subjects’ average spectrum scenarios were merged to obtain a grand-average spectrum corresponding to preferred and non-preferred musical stimuli (see Fig. 2d).

### Results of Experiment 1

#### Aesthetic appreciation of musical stimuli

The aesthetic ratings associated with subjectively preferred (mean = 7.393; SD = 0.961) versus non-preferred (mean = 4.683; SD = 1.249) musical stimuli were significantly different (t_17_ = -13.43; p < 0.001; Cohen’s *d* = 2.432) as revealed by the two-tailed point by point t-test. The comparison of aesthetic ratings corresponding to musical stimuli with different objective features (key, tempo, direction), revealed that, as expected, only the direction of presentation (forward vs. backward) significantly affected aesthetic rating (t_17_ = 4.99; p < 0.001; Cohen’s *d* = 1.407), with higher ratings for forward (mean = 6.967; SD = 1.242) compared to backward music (mean = 5.18; SD = 1.298). Preferences were neither significantly affected by different keys (MINOR: mean = 6.298; SD = 1.223; MAJOR: mean = 5.848; SD = 1.045; t_17_ = -1.882; p = 0.077; Cohen’s *d* = 1.014) nor by different tempo (SLOW: mean = 6.086; SD = 1.119; FAST: mean = 6.06; SD = 1.115; t_17_ = 0.121; p = 0.905; Cohen’s *d* = 0.922). Therefore, subsequent control analyses run to exclude the possibility that electrophysiological results were affected by mere objective features of musical stimuli rather than by subjective preferences were restricted to the comparison of results following forward and backward music listening.

#### MMN results

Point-by-point group level analyses on single subjects’ MMN differential waves revealed the following results. To exclude that the effect on perceptual learning was merely due to the fact that musical pieces were played forward or backwards, we compared MMN responses registered after forward versus backward music listening. This analysis revealed no significant difference between MMN registered after forward versus backward music listening (no cluster survived permutation correction). Crucially, once MMN were grouped according to subjective aesthetic preferences (preferred vs. non-preferred), the same analysis highlighted several clusters of significance: the point-by-point t-test comparing MMN registered after preferred versus non-preferred music listening revealed a large significant cluster extending across frontal electrodes from 100 to 276 ms post-onset and including the MMN negative peak at 120–150 ms post-onset (Fig. 2b). The effect showed its maximum over F3 and F7 and remained significant over fronto-lateral electrodes during the entire time cluster (see scalpmaps in Fig. 2b). A second large frontal cluster (Fpz, Fp1, Fp2, F7, F3, Fz, F4, F8, Fc5, Fc1, Fc2, Fc6, Fcz), centred around 305–364 ms post-onset, corresponded to the latency of the offset of the P3 component and the onset of the N400 component.

#### Trial-by-trial correlation with Bayesian Surprise

In order to test for the presence of a possible enhancement of the encoding of sensory surprise (i.e., informative value) after preferred music listening, compared to non-preferred music listening, we performed a point-by-point correlation analysis between trial-by-trial fluctuations and Bayesian Surprise, an information-theoretic index of sensory surprise and EEG amplitudes (see Methods and OSM). In agreement with previous studies (Mousavi et al., 2020; Rabovsky et al., 2018), Bayesian Surprise was more strongly correlated with trial-by-trial amplitude fluctuations in correspondence of the MMN peak around 130 ms post-onset (as indicated by r-values local peaks; Fig. 2c), the P300 peak at 230 ms post-onset, and the N400 negative component (395 ms post-onset). A single significant cluster, however, survived permutation cluster-based correction in the point-by-point t-test comparing r-values corresponding to preferred versus non-preferred music listening. This cluster was centred over frontal electrodes (F7, F3, Fz, Fc1, Fc2) at 356–396 ms post-onset and corresponded to the latency and scalp amplitude distribution of the N400 component (Fig. 2c).

#### Results in the frequency domain

Additionally, to explore possible differences in resting state brain oscillatory activity following preferred versus non-preferred music listening, we computed, for each participant, the average power spectrum registered during the last 2 min of passive music (or reversed music) listening. For each participant and for each channel, we obtained two curves (preferred vs. non-preferred music) indicating the average power across frequencies (see grand-average values in Fig. 2d). These data served as the input for a subsequent group level point-by-point t-test comparing the distribution of power across frequencies following preferred and non-preferred music listening. In accordance with previous findings (Jaušovec et al., 2006; Verrusio et al., 2015), this analysis revealed a significant increase in the power of the posterior upper alpha rhythm following preferred versus non-preferred music listening (see Fig. 2d). As reported in Fig. 2d, the point-by-point t-test revealed a significant cluster surviving permutation cluster-based correction, corresponding to the upper alpha band (10.286–11.067 Hz), peaking at 10.5 Hz and centred over centroparietal electrodes (CP1, CP2, Pz, P4). As can be seen in Fig. 2d, this analysis revealed a change in the spectral profile of the alpha frequency, with increased power in the upper alpha (10–13 Hz), compared to the lower alpha (8–10 Hz), after listening to preferred music.

In sum, we performed Experiment 1 to investigate the neural attunement to sensory surprise after listening to subjectively preferred versus non-preferred music. An increased correlation between surprise and EEG responses, also evidenced by larger MMN responses, after preferred music listening demonstrates the possible presence of a positive aftereffect on perceptual learning dynamics triggered by subjective musical aesthetic pleasure. The aftereffect is preceded by a power shift toward upper alpha frequencies during preferred music listening. Such an increase in upper alpha power is generally related to increased attention to external auditory stimuli (Sadaghiani et al., 2010; see Discussion). To further evaluate the effect of musical preferences on attentional dynamics (toward external stimuli and toward the self) we designed Experiment 2.

## Experiment 2

We predicted that the enhancement of participants’ attention toward the sensory environment, resulting in an amplified perceptual learning, may be realized at the expense of self-focused attention. To verify the hypothesis that aesthetic pleasure redirects attentional resources from self-related stimuli to environmental stimuli, we designed Experiment 2, which exploits the well-acknowledged link between performances in HBD tasks and self-directed attentional focus (Pollatos et al., 2016; see also the Discussion).

### Methods

#### Participants

Twenty healthy right-handed subjects participated in the experiment (16 women, mean age: 25.500 years; SD: ± 2.212; mean years of education: 18.550; SD: ± 1.050). This group of participants was entirely different from the sample of Experiment 1 (i.e., participants of Experiment 1 did not take part in Experiment 2). All participants gave their written informed consent to participate in the study, which conformed to the standards required by the Declaration of Helsinki and was approved by the Ethics Committee of the University of Turin (Prot. n. 121724 – 01/03/18). Participants were not compensated for taking part in the experiment.

Sample size (N = 20) was a priori determined through a power analysis based on the effect size obtained in a pilot experiment identical to this behavioural experiment, involving ten additional participants and comparing HBD accuracies (ACC) between preferred and non-preferred conditions (Cohen’s *d* = 0.613; α = 0.05; required power = 0.95).

#### Stimuli and experimental design

##### Procedures

Similarly to Experiment 1, Experiment 2 was based on a within-subject design and consisted of eight blocks of three consecutive tasks: (1) passive listening to musical pieces (i.e., ‘Musical Listening’ as in Experiment 1); (2) an Aesthetic Judgement task identical to Experiment 1; (3) an active heartbeat counting (i.e. ‘Counting Task’) in which participants were asked to estimate the number of heartbeats they felt during different time intervals (Ainley & Tsakiris, 2013; Koeppel et al., 2020), while their effective heartbeat was recorded using an ad hoc device (Astel Electronic Engineering Srl, Torino, Italy). Musical stimuli, passive musical listening and aesthetic judgements procedures were identical to Experiment 1.

##### Counting task

Participants were asked to lie down after having listened to and judged each musical excerpt. After the aesthetic judgment was collected, the Counting Task began. In each block subjects were asked to silently count the number of heartbeats during four time intervals. To avoid participants using temporal cues during beat estimation, the duration of the four intervals varied: intervals of 25, 30, 35 and 45 s were used, in accordance with the Mental Tracking Method described by Schandry (1981). The order of presentation of the different time intervals employed in the counting tasks was fully randomised across blocks. The beginning and the end of each interval in the counting phase were signalled vocally by the experimenter. After the stop signal, for each interval, subjects were required to verbally report the number of heartbeats they counted. Subjects were informed about neither the length of the counting intervals nor their performance.

### Data analysis

#### Aesthetic judgements

Analyses on aesthetic ratings were the same as those employed in Experiment 1.

#### HBD accuracy

ECG raw data were analysed with the Signal Processing Toolbox in MATLAB. Low (45 Hz) and high (0.5 Hz) pass filters were applied to the signal to filter out noise. The number of R-wave peaks on the ECG trace for each interval in each block was then measured and recorded. ECG recordings were visually inspected for artifacts and the number of R-wave peaks was recounted manually, when necessary.

Interoceptive accuracy was derived according to Schandry (1981) as the difference between reported and objective (obtained from the ECG) heart beats, divided by the objective number of heart beats. This index is thus inversely related to HBD accuracy. Resulting accuracy scores were averaged across the four intervals, yielding an average value for each block/musical stimulus per each participant. Average accuracies were grouped according to the subjective appreciation rating collected after each musical excerpt: one group was composed by the accuracies corresponding to the four most appreciated musical pieces, while the accuracies corresponding to the remaining four least appreciated pieces were assigned to the non-preferred group. A two-tailed t-test was performed to assess any significant difference in accuracies between the two conditions. Similar control analyses were conducted for the mean heart rate recorded during the listening phase.

### Results of Experiment 2

#### Aesthetic appreciation of musical stimuli

As in Experiment 1, the aesthetic ratings associated with subjectively preferred (mean = 7.519; SD = 0.705) versus non-preferred (mean = 5.086; SD = 1.322) musical stimuli were significantly different (t_19_ = 12.99; p < 0.001; Cohen’s *d* = 2.906) as revealed by the two-tailed point-by-point t-test. The direction of presentation of musical stimuli also affected AJs, with significantly higher aesthetic ratings for forward music (mean = 7.065; SD = 0.894) as compared to backward music (mean = 5.527; SD = 1.448; t_19_ = 4.816; p < 0.001; Cohen’s *d* = 1.077). Similarly, mean appreciation was also significantly higher for minor key music (mean = 6.665; SD = 1.018) as compared to major key music (mean = 5.927; SD = 1.074; t_19_ = 4.189; p < 0.001; Cohen’s *d* = 0.937), suggesting that AJs were also influenced by key. The comparison between AJs corresponding to fast versus slow tempo music revealed no significant difference (t_19_ = 0.384; p = 0.705; Cohen’s *d* = 0.086). Coherently, direction and key were included as control factors in subsequent analyses (to verify whether HBD accuracies were modulated by these features).

#### HBD accuracy

**Fig. 3:**
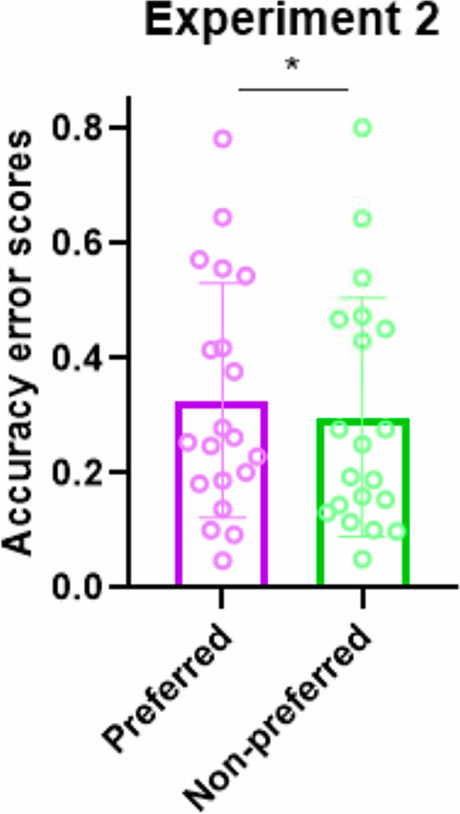
Experiment 2 – results. The histogram represents average error scores (the index is inversely related to heart-beat detection (HBD) accuracies; see Methods) from the HBD task following preferred and non-preferred music listening. Dots represent single subjects’ error scores. Bars represent standard deviations

To evaluate the effect of subjective preferences on HBD accuracies, as in Experiment 1, results were split into two conditions (Preferred and Non-preferred) according to subjective preferences: for each subject separately, HBD accuracy scores registered after listening to the four least appreciated musical stimuli were averaged together and corresponded to the non-preferred condition, while the remaining four most appreciated musical stimuli corresponded to the preferred condition. On average, as represented in Fig. 3, HBD accuracies were lower in the preferred condition (grand average (N = 20) = 0.327; SD = 0.204) compared to the non-preferred one (grand average (N = 20) = 0.298; SD = 0.208). The group-level two-tailed t-test comparing HBD accuracies revealed a significant difference between the preferred and non-preferred conditions (t_19_ = 2.87; p = 0.01; Cohen’s *d* = 0.141). To exclude the possibility that this result was influenced by heart rate variability across preference conditions, we measured the pulse for preferred and non-preferred music. Heart rate was comparable after listening to preferred (mean = 72.526; SD = 10.347) and non-preferred (mean = 70.773; SD = 8.865) music, with no significant difference between the two (t_19_ = 1.25; p = 0.227; Cohen’s *d* = 0.274).

Additionally, as a further control, to exclude the potential effect of music stimuli basic features, we compared ACC collected after listening to forward versus backward music and after listening to major versus minor key music, independently from subjective aesthetic preferences. Different to the modulatory effect of aesthetic preferences, ACCs in the HBD task were significantly modulated neither by the direction of presentation (forward vs. backwards: t_19_ = 0.533; p = 0.596; Cohen’s *d* = 0.06), nor by the musical key (major vs. minor: t_19_ = 1.747; p = 0.085; Cohen’s *d* = 0.195).

## Discussion

In accordance with our initial hypotheses, after listening to preferred music compared to non-preferred music: (1) MMN responses to frequency deviant sounds were larger; (2) the correlation between single-trial N400 amplitudes and Bayesian Surprise was stronger; (3) resting-state upper alpha power was increased; (4) HBD accuracy was lower.

The magnitude of MMN responses is considered to reflect the update of brain predictive representations triggered by surprising sensory inputs (i.e., perceptual learning; Ostwald et al., 2012; Rosch et al., 2019). Therefore, we demonstrated the presence of an aftereffect on perceptual learning triggered by subjective musical preferences. This is further confirmed by the enhanced correlation between N400 amplitudes and Bayesian Surprise, suggesting a greater attunement of the sensory system to changes occurring in the sensory environment. The N400, as the MMN, is thought to reflect the amount of new information that is conveyed by an incoming unpredicted stimulus (Kuperberg, 2016). Accordingly, the N400 brainwave is sensitive to violations of global regularities in repetitive auditory stimulation (Liaukovich et al., 2020). On the other hand, results of HBD accuracies indicate a diminished self-focused attention. Indeed, it is well known that self-focused attention modulates interoceptive accuracy in HBD tasks (Pollatos et al., 2016). As an example, HBD accuracies can be enhanced by viewing one’s reflection in the mirror (Ainley et al., 2012; Weisz et al., 1988), or one’s own photograph (Maister & Tsakiris, 2014), or words that are related to the self (Ainley & Tsakiris, 2013).

Altogether, in agreement with previous accounts of aesthetic appreciation (Sarasso, et al., 2020a), our findings suggest that aesthetic appreciation of music can enhance low-level perceptual learning via the redirection of processing resources from self-focused attention to environmental stimuli, minutes after the aesthetic experience is over. To our knowledge, our study provides the first empirical evidence of a long-lasting learning-oriented attentional modulation induced by aesthetic appreciation. These results confirm previous theoretical predictions suggesting that the physiological, cognitive and affective states characterising aesthetic appreciations do not only depend on the bottom-up processing of beautiful sensory features, but can also be induced by the top-down attitudinal (Stolnitz, 1978), task-related (Jacobsen et al., 2006) or contextual (Pelowski et al., 2017) expectations of the beholder (i.e., the expectancy of an aesthetically pleasurable sensory context). In our study, the beauty-induced effect on perceptual sensitivity might be mediated by a top-down attentional up-weighting of sensory information (Sarasso, et al., 2020a), here referred to as aesthetic attitude. Indeed, attention can be operationalized as the responsiveness (i.e., sensitivity) of sensory cortices to sensory surprise driven by the plastic modulation of the synaptic gain in primary sensory cortices (Brown & Friston, 2012). The correlation between brainwaves and Bayesian Surprise measured in Experiment 1 ideally captures such neural dynamics, since it measures the extent to which sensory cortices respond to the information conveyed by sensory stimuli (Ostwald et al., 2012; Sarasso et al., 2021b). Therefore, in metaphorical terms, aesthetic appreciation induced by music might act as a sort of “valve” that turns up the volume of the sensory environment at the expense of self-focused attention.

This interpretation fits well with recent theories suggesting that aesthetic appreciation might be considered as a hedonic feedback (Schoeller & Perlovsky, 2016; Van de Cruys & Wagemans, 2011) on information gains (Gottlieb et al., 2013; Kaplan & Oudeyer, 2004), able to assign value to information and to further direct the sampling of sensory inputs (Sarasso et al., 2020a). The aesthetic attentional attitude might represent the long-lasting effect of this greater attunement with perceptual experiences.

Furthermore, we propose that the modulations in EEG alpha powers might represent a possible neural signature underlying the aesthetic attitude. The alpha frequency spectral profile at rest is an effective marker of subjects’ state of arousal and attention. A change in the spectral profile toward the upper alpha band (10–13 Hz) was found to be associated with task performance and cognitive abilities (Angelakis et al., 2007; Klimesch, 1997; Richard Clark et al., 2004), reaction time and speed of information processing (Klimesch et al., 1996) in oddball (Gath et al., 1983) and detection tasks (Lockley et al., 2006) and the maintenance of auditory attention (Dockree et al., 2007). Coherently, the correlation between alpha spectral power shift toward the upper alpha frequency band (10–13 Hz) and cerebral blood flow – which indicates a high baseline energy metabolism associated with increased attention to external stimuli (Jann et al., 2010) – is associated with a cortico-thalamic network of brain areas controlling the modulation of attention and preparedness (Alper et al., 2006; Jann et al., 2010). Similarly, the activity in a cingulo-insular-thalamic network responsible for sustained alertness and attention to external stimuli is positively correlated with the global field power of oscillations in the upper alpha band (Sadaghiani et al., 2010). Conversely, upper alpha frequency at rest was shown to be negatively correlated with activations in most brain areas included in the default mode network (Bowman et al., 2017). In sum, upper alpha power is generally regarded as a neural signature of tonic alertness and sustained attention to external auditory stimuli (see Sadaghiani et al., 2010, for a review). Namely, upper alpha might enhance sensitivity by rhythmically clearing the flood of sensory information on a rapid time scale to reduce distraction and hence enhance detection of novel (i.e., surprising) sensory information (Sadaghiani et al., 2010). This fits well with the evidence of enhanced MMN responses to surprising stimuli after more appreciated music and with the hypothesized role of beauty-induced alertness in triggering cognitive enhancements following music listening (Thompson et al., 2001). Together with our results on HBD accuracies, increased upper alpha power might therefore indicate a redistribution of attentional resources from self-focused perception toward novel environmental auditory stimuli following preferred music listening.

To conclude, we suggest that musical aesthetic experiences might trigger the attentional up-weighting of external sensory stimuli at the expense of self-focused attention. These results, if confirmed by future studies, suggest that aesthetic experiences could be employed as an original factor for the study of the neurocognitive mechanisms associated with learning and memory retrieval (Lehmann & Seufert, 2018). Moreover, the role of aesthetic experiences in automatically guiding attentional processes toward learning and change has potentially interesting applications in numerous human activities, for example teaching (Mastandrea et al., 2019), neurorehabilitation and psychotherapy (Beebe, 2010; Roubal et al., 2017; Spagnuolo Lobb, 2018).

## Supplementary Information

ESM 1 (PDF 185 kb)

## References

[CR1] Ainley V, Tsakiris M (2013). Body conscious? Interoceptive awareness, measured by heartbeat perception, is negatively correlated with self-objectification. PLoS ONE.

[CR2] Ainley V, Tajadura-Jiménez A, Fotopoulou A, Tsakiris M (2012). Looking into myself: Changes in interoceptive sensitivity during mirror self-observation. Psychophysiology.

[CR3] Alper, K. R., John, E. R., Brodie, J., Günther, W., Daruwala, R., & Prichep, L. S. (2006). Correlation of PET and qEEG in normal subjects. *Psychiatry Research - Neuroimaging*. 10.1016/j.pscychresns.2005.06.008 10.1016/j.pscychresns.2005.06.008 16603341

[CR4] Angelakis E, Stathopoulou S, Frymiare JL, Green DL, Lubar JF, Kounios J (2007). EEG neurofeedback: A brief overview and an example of peak alpha frequency training for cognitive enhancement in the elderly. The Clinical Neuropsychologist.

[CR5] Baldeweg T, Klugman A, Gruzelier J, Hirsch SR (2004). Mismatch negativity potentials and cognitive impairment in schizophrenia. Schizophrenia Research.

[CR6] Baldi P, Itti L (2010). Of bits and wows: A Bayesian theory of surprise with applications to attention. Neural Networks.

[CR7] Beebe, J. (2010). Psychotherapy in the aesthetic attitude. *Journal of Analytical Psychology*. 10.1111/j.1468-5922.2010.01835.x 10.1111/j.1468-5922.2010.01835.x 20518960

[CR8] Bowman, A. D., Griffis, J. C., Visscher, K. M., Dobbins, A. C., Gawne, T. J., DiFrancesco, M. W., & Szaflarski, J. P. (2017). Relationship between alpha rhythm and the default mode network: An EEG-fMRI study. *Journal of Clinical Neurophysiology*. 10.1097/WNP.0000000000000411 10.1097/WNP.0000000000000411 PMC8428580 28914659

[CR9] Brewer R, Murphy J, Bird G (2021). Atypical interoception as a common risk factor for psychopathology: A review. Neuroscience and Biobehavioral Reviews.

[CR10] Brown HR, Friston KJ (2012). Dynamic causal modelling of precision and synaptic gain in visual perception - an EEG study. NeuroImage.

[CR11] Bruno V, Ronga I, Fossataro C, Galigani M, Sacco K, Garbarini F (2020). Long-term limb immobilization modulates inhibition-related electrophysiological brain activity. NeuroImage.

[CR12] Cover, T. M., & Thomas, J. A. (1991). Elements of information theory. In *Elements of information theory*. 10.1002/0471200611

[CR13] Dockree PM, Kelly SP, Foxe JJ, Reilly RB, Robertson IH (2007). Optimal sustained attention is linked to the spectral content of background EEG activity: Greater ongoing tonic alpha (∼10 Hz) power supports successful phasic goal activation. European Journal of Neuroscience.

[CR14] Faraji, M., Preuschoff, K., & Gerstner, W. (2018). Balancing new against old information: The role of puzzlement surprise in learning. *Neural Computation*. 10.1162/NECO_a_01025 10.1162/neco_a_01025 29064784

[CR15] Freyer, F., Becker, R., Dinse, H. R., & Ritter, P. (2013). State-dependent perceptual learning. *Journal of Neuroscience*. 10.1523/JNEUROSCI.4039-12.2013 10.1523/JNEUROSCI.4039-12.2013 PMC6619196 23407948

[CR16] Fritz TH, Schmude P, Jentschke S, Friederici AD, Koelsch S (2013). From understanding to appreciating music cross-culturally. PLoS ONE.

[CR17] García-Cordero I, Esteves S, Mikulan EP, Hesse E, Baglivo FH, Silva W, García M, Vaucheret E, Ciraolo C, García HS, Adolfi F, Pietto M, Herrera E, Legaz A, Manes F, García AM, Sigman M, Bekinschtein TA, Ibáñez A, Sedeño L (2017). Attention, in and out: Scalp-level and Intracranial EEG correlates of interoception and exteroception. Frontiers in Neuroscience.

[CR18] Garrido MI, Teng CLJ, Taylor JA, Rowe EG, Mattingley JB (2016). Surprise responses in the human brain demonstrate statistical learning under high concurrent cognitive demand. Npj Science of Learning.

[CR19] Gath I, Lehmann D, Bar-on E (1983). Fuzzy clustering of eeg signal and vigilance performance. International Journal of Neuroscience.

[CR20] Gottlieb J, Oudeyer PY, Lopes M, Baranes A (2013). Information-seeking, curiosity, and attention: Computational and neural mechanisms. Trends in Cognitive Sciences.

[CR21] Harris A, Hare T, Rangel A (2013). Temporally dissociable mechanisms of self-control: Early attentional filtering versus late value modulation. Journal of Neuroscience.

[CR22] Jacobsen T, Schubotz RI, Höfel L, Cramon DYV (2006). Brain correlates of aesthetic judgment of beauty. NeuroImage.

[CR23] Jann, K., Koenig, T., Dierks, T., Boesch, C., & Federspiel, A. (2010). Association of individual resting state EEG alpha frequency and cerebral blood flow. *NeuroImage.*10.1016/j.neuroimage.2010.02.024 10.1016/j.neuroimage.2010.02.024 20156573

[CR24] Jaušovec, N., Jaušovec, K., & Gerlič, I. (2006). The influence of Mozart’s music on brain activity in the process of learning. *Clinical Neurophysiology*. 10.1016/j.clinph.2006.08.010 10.1016/j.clinph.2006.08.010 17029951

[CR25] Jenkins JS (2001). The Mozart effect. Journal of the Royal Society of Medicine.

[CR26] Jung TP, Makeig S, Humphries C, Lee TW, Mckeown MJ, Iragui V, Sejnowski TJ (2000). Removing electroencephalographic artifacts by blind source separation. Psychophysiology.

[CR27] Kaplan, F., & Oudeyer, P.-Y. (2004). Maximizing learning progress: An internal reward system for development. *Embodied Artificial Intelligence.*10.1007/b99075

[CR28] Kemp, G. (1999). The aesthetic attitude. *British Journal of Aesthetics, 39*(4).

[CR29] Klimesch W (1997). EEG-alpha rhythms and memory processes. International Journal of Psychophysiology: Official Journal of the International Organization of Psychophysiology.

[CR30] Klimesch W, Doppelmayr M, Schimke H, Pachinger T (1996). Alpha frequency, reaction time, and the speed of processing information. Journal of Clinical Neurophysiology: Official Publication of the American Electroencephalographic Society.

[CR31] Koeppel CJ, Ruser P, Kitzler H, Hummel T, Croy I (2020). Interoceptive accuracy and its impact on neuronal responses to olfactory stimulation in the insular cortex. Human Brain Mapping.

[CR32] Kuperberg GR (2016). Separate streams or probabilistic inference? What the N400 can tell us about the comprehension of events. Language, Cognition and Neuroscience.

[CR33] Lehmann, J. A. M., & Seufert, T. (2018). Can music foster learning - Effects of different text modalities on learning and information retrieval. *Frontiers in Psychology*. 10.3389/fpsyg.2017.02305 10.3389/fpsyg.2017.02305 PMC5767298 29375429

[CR34] Liaukovich K, Ukraintseva Y, Martynova O (2020). Implicit auditory perception of local and global irregularities in passive listening condition. BioRxiv.

[CR35] Lieder, F., Daunizeau, J., Garrido, M. I., Friston, K. J., & Stephan, K. E. (2013). Modelling trial-by-trial changes in the mismatch negativity. *PLoS Computational Biology, 9*(2). 10.1371/journal.pcbi.1002911 10.1371/journal.pcbi.1002911 PMC3578779 23436989

[CR36] Lockley SW, Evans EE, Scheer FAJL, Brainard GC, Czeisler CA, Aeschbach D (2006). Short-wavelength sensitivity for the direct effects of light on alertness, vigilance, and the waking electroencephalogram in humans. Sleep.

[CR37] Maister L, Tsakiris M (2014). My face, my heart: Cultural differences in integrated bodily self-awareness. Cognitive Neuroscience.

[CR38] Maris, E., & Oostenveld, R. (2007). Nonparametric statistical testing of EEG- and MEG-data. *Journal of Neuroscience Methods*. 10.1016/j.jneumeth.2007.03.024 10.1016/j.jneumeth.2007.03.024 17517438

[CR39] Mastandrea, S., Fagioli, S., & Biasi, V. (2019). Art and psychological well-being: Linking the brain to the aesthetic emotion. *Frontiers in Psychology*. 10.3389/fpsyg.2019.00739 10.3389/fpsyg.2019.00739 PMC6458291 31019480

[CR40] Menninghaus W, Wagner V, Wassiliwizky E, Schindler I, Hanich J, Jacobsen T, Koelsch S (2019). What are aesthetic emotions?. Psychological Review.

[CR41] Molholm, S., Martinez, A., Ritter, W., Javitt, D. C., & Foxe, J. J. (2005). The neural circuitry of pre-attentive auditory change-detection: An fMRI study of pitch and duration mismatch negativity generators. *Cerebral Cortex*. 10.1093/cercor/bhh155 10.1093/cercor/bhh155 15342438

[CR42] Mousavi, Z., Kiani, M. M., & Aghajan, H. (2020). Brain signatures of surprise in EEG and MEG data. *BioRxiv*, 2020.01.06.895664. 10.1101/2020.01.06.895664

[CR43] Näätänen, R., Gaillard, A. W. K., & Mäntysalo, S. (1978). Early selective-attention effect on evoked potential reinterpreted. *Acta Psychologica*. 10.1016/0001-6918(78)90006-9 10.1016/0001-6918(78)90006-9 685709

[CR44] Näätänen R, Paavilainen P, Rinne T, Alho K (2007). The mismatch negativity (MMN) in basic research of central auditory processing: A review. Clinical Neurophysiology.

[CR45] Nanay B (2016). *Aesthetics as philosophy of perception*.

[CR46] Novembre, G., Pawar, V., Bufacchi, R., Kilintari, M., Srinivasan, M., Rothwell, J., Haggard, P., & Iannetti, G. (2018). Saliency detection as a reactive process: Unexpected sensory events evoke cortico-muscular coupling. *The Journal of Neuroscience*. 10.1523/JNEUROSCI.2474-17.2017 10.1523/JNEUROSCI.2474-17.2017 PMC5830523 29378865

[CR47] Ostwald D, Spitzer B, Guggenmos M, Schmidt TT, Kiebel SJ, Blankenburg F (2012). Evidence for neural encoding of Bayesian surprise in human somatosensation. NeuroImage.

[CR48] Pearce, M. T., Zaidel, D. W., Vartanian, O., Skov, M., Leder, H., Chatterjee, A., & Nadal, M. (2016). Neuroaesthetics: The cognitive neuroscience of aesthetic experience. *Perspectives on Psychological Science*. 10.1177/1745691615621274 10.1177/1745691615621274 26993278

[CR49] Pelowski, M., Markey, P. S., Forster, M., Gerger, G., & Leder, H. (2017). Move me, astonish me… delight my eyes and brain: The Vienna Integrated Model of top-down and bottom-up processes in Art Perception (VIMAP) and corresponding affective, evaluative, and neurophysiological correlates. *Physics of Life Reviews*. 10.1016/j.plrev.2017.02.003 10.1016/j.plrev.2017.02.003 28347673

[CR50] Pollatos O, Herbert BM, Berberich G, Zaudig M, Krauseneck T, Tsakiris M (2016). Atypical self-focus effect on interoceptive accuracy in Anorexia Nervosa. Frontiers in Human Neuroscience.

[CR51] Rabovsky M, Hansen SS, McClelland JL (2018). Modelling the N400 brain potential as change in a probabilistic representation of meaning. Nature Human Behaviour.

[CR52] Rauscher FH, Shaw GL, Ky CN (1993). Music and spatial task performance. Nature.

[CR53] Richard Clark C, Veltmeyer MD, Hamilton RJ, Simms E, Paul R, Hermens D, Gordon E (2004). Spontaneous alpha peak frequency predicts working memory performance across the age span. International Journal of Psychophysiology: Official Journal of the International Organization of Psychophysiology.

[CR54] Ronga, I., Valentini, E., Mouraux, A., & Iannetti, G. D. (2013). Novelty is not enough: Laser-evoked potentials are determined by stimulus saliency, not absolute novelty. *Journal of Neurophysiology*. 10.1152/jn.00464.2012 10.1152/jn.00464.2012 PMC3567386 23136349

[CR55] Rosch RE, Auksztulewicz R, Leung PD, Friston KJ, Baldeweg T (2019). Selective prefrontal disinhibition in a roving auditory oddball paradigm under N-Methyl-D-Aspartate receptor blockade. Biological Psychiatry: Cognitive Neuroscience and Neuroimaging.

[CR56] Roubal, J., Francesetti, G., & Gecele, M. (2017). Aesthetic diagnosis in gestalt therapy. *Behavioral Science*. 10.3390/bs7040070 10.3390/bs7040070 PMC5746679 29039752

[CR57] Sadaghiani, S., Scheeringa, R., Lehongre, K., Morillon, B., Giraud, A. L., & Kleinschmidt, A. (2010). Intrinsic connectivity networks, alpha oscillations, and tonic alertness: A simultaneous electroencephalography/functional magnetic resonance imaging study. *Journal of Neuroscience*. 10.1523/JNEUROSCI.1004-10.2010 10.1523/JNEUROSCI.1004-10.2010 PMC6633365 20668207

[CR58] Sarasso P, Ronga I, Pistis A, Forte E, Garbarini F, Ricci R, Neppi-Modona M (2019). Aesthetic appreciation of musical intervals enhances behavioural and neurophysiological indexes of attentional engagement and motor inhibition. Scientific Reports.

[CR59] Sarasso, P., Neppi-Modona, M., Sacco, K., & Ronga, I. (2020a). “Stopping for knowledge”: The sense of beauty in the perception-action cycle. *Neuroscience & Biobehavioral Reviews*. 10.1016/j.neubiorev.2020.09.004 10.1016/j.neubiorev.2020.09.004 32926914

[CR60] Sarasso P, Ronga I, Kobau P, Bosso T, Artusio I, Ricci R, Neppi-Modona M (2020). Beauty in mind: Aesthetic appreciation correlates with perceptual facilitation and attentional amplification. Neuropsychologia.

[CR61] Sarasso, P., Perna, P., Barbieri, P., Neppi-Modona, M., Sacco, K., & Ronga, I. (2021a). Memorisation and implicit perceptual learning are enhanced for preferred musical intervals and chords. *Psychonomic Bulletin & Review*. 10.3758/s13423-021-01922-z 10.3758/s13423-021-01922-z PMC8500890 33945127

[CR62] Sarasso, P., Neppi-Modona, M., Rosaia, N., Perna, P., Barbieri, P., Del Fante, E., Sacco, K., & Ronga, I. (2021b). Nice and easy: Mismatch negativity responses reveal a significant correlation between aesthetic appreciation and perceptual learning. *Journal of Experimental Psychology – General*. 10.1037/xge0001149 10.1037/xge0001149 34793192

[CR63] Schandry R (1981). Heart beat perception and emotional experience. Psychophysiology.

[CR64] Schoeller, F., & Perlovsky, L. (2016). Aesthetic chills: Knowledge-acquisition, meaning-making, and aesthetic emotions. *Frontiers in Psychology*, *7*(AUG). 10.3389/fpsyg.2016.01093 10.3389/fpsyg.2016.01093 PMC4973431 27540366

[CR65] Sigala R, Haufe S, Roy D, Dinse H, Ritter P (2014). The role of alpha-rhythm states in perceptual learning: Insights from experiments and computational models. Frontiers in Computational Neuroscience.

[CR66] Spagnuolo Lobb, M. (2018). Aesthetic relational knowledge of the field: A revised concept of awareness in gestalt therapy and contemporary psychiatry. *Gestalt Review*. 10.5325/gestaltreview.22.1.0050

[CR67] Stolnitz, J. (1978). “The aesthetic attitude” in the rise of modern aesthetics. *The Journal of Aesthetics and Art Criticism.*10.2307/430481

[CR68] Thompson WF, Schellenberg EG, Husain G (2001). Arousal, mood, and the Mozart effect. Psychological Science.

[CR69] Van de Cruys S, Wagemans J (2011). Putting reward in art: A tentative prediction error account of visual art. I-Perception.

[CR70] Vassiliou F (2020). Aesthetic disinterestedness in neuroaesthetics: A phenomenological critique. Aesthetic Investigations.

[CR71] Verrusio, W., Ettorre, E., Vicenzini, E., Vanacore, N., Cacciafesta, M., & Mecarelli, O. (2015). The Mozart effect: A quantitative EEG study. *Consciousness and Cognition*. 10.1016/j.concog.2015.05.005 10.1016/j.concog.2015.05.005 26036835

[CR72] Weisz J, Bálazs L, ÁDám G (1988). The influence of self-focused attention on heartbeat perception. Psychophysiology.

