# Supplementary material for: Preferred music listening is associated with perceptual learning enhancement at the expense of self-focused attention
Source: Psychon Bull Rev. 2022 Jun 6;29(6):2108–21. doi: 10.3758/s13423-022-02127-8 (PMC9722857; doi:10.3758/s13423-022-02127-8)
Supplement: Supplementary file 1 — (PDF 185 kb) [file 13423_2022_2127_MOESM1_ESM.pdf]

Supplementary Information

Bayesian Surprise

We compute the Bayesian surprise for each trial under a Beta-Binomial model of Bayesian learning of the stimulus probabilities (Ostwald et al., 2012). The model assumes that the brain implements a sequential learning procedure starting from an uninformative prior, updates this prior according to the subsequent observations, and computes Bayesian surprise as the Kullback-Leibler divergence between prior and posterior (Ostwald et al., 2012). Following Otswald et al. (2012), we use a variant of this model that assumes an exponential forgetting of stimuli which are observed in the distant past.

Formally, we assume that the probability of observing a low ($x = 0$) or high ($x = 1$) intensity stimulus at a given trial is described by a Bernoulli distribution with parameter $\mu \in [0, 1]$:

$$\Pr\left(x|\mu\right) = \mu^x \left(1-\mu\right)^{1-x}, \, x \in \{0,1\}.$$

The true value of μ is unknown by the subject, and the initial uncertainty is modelled by means of an informative prior of type Beta, whose density function is uniform on the unit interval:

$$f\left(\mu\right) = 1, \, \forall \mu \in [0,1].$$

On each trial, the prior is sequentially updated according to the observed data likelihood to form a posterior distribution over μ. After N trials, let $\boldsymbol{x}^N$ denote the sequence of observed stimuli

$$\boldsymbol{x}^N = \begin{bmatrix} x_1 \\ \vdots \\ x_N \end{bmatrix}$$

where $x_i \in \{0, 1\}$ for each $i = 1, ..., N$. Under standard Bayesian learning, the posterior over μ is computed as follows. The probability of observing a $\boldsymbol{x}^N$ when the true parameter is μ is:

$$\Pr\left(\boldsymbol{x}^N|\mu\right) = \mu^{n_N} \left(1-\mu\right)^{m_N}.$$

Bayes rule implies that the posterior over μ is a Beta distribution with density

$$f\left(\mu|\boldsymbol{x}^N\right) = \frac{\Pr\left(\boldsymbol{x}^N|\mu\right) f\left(\mu\right)}{\int_0^1 \Pr\left(\boldsymbol{x}^N|\nu\right) f\left(\nu\right) \mathrm{d}\nu} = \frac{\mu^{n_N}\left(1-\mu\right)^{m_N}}{B\left(1+n_N, 1+m_N\right)}$$

where $n_N = |\{i : x_i = 1\}|$ is the nuber of high-intensity stimuli, $m_N = N - n_N$ is the number of low-intensity stimuli and B is the Beta function (Ostwald et al., 2012).

In order to account for a forgetting dynamics, instead of using the accumulative stimulus counts n_N and m_N in previous formula, the model employed here weights past observations according to an exponential function. Define the weighted stimulus counts n_N^τ and m_N^τ by

$$n_N^\tau := \sum_{i=1}^{N} \exp\left(-\frac{1}{\tau}\left(N-i\right)\right) x_i$$

and

$$m_N^\tau := \sum_{i=1}^{N} \exp\left(-\tau\left(N-i\right)\right)\left(1-x_i\right).$$

$\tau \geq 0$ is a parameter governing the forgetting dynamics: for $\tau = 0$ we have $n_N^\tau, m_N^\tau = n_N, m_N$, whereas increasing the value of τ implies that past observations are weighted less and less. This results in a posterior density given by

$$f^\tau\left(\mu|\boldsymbol{x}^N\right) = \frac{\mu^{n_N^\tau}\left(1-\mu\right)^{m_N^\tau}}{B\left(1+n_N^\tau, 1+m_N^\tau\right)}.$$

Finally, the model quantifies the degree of learning as the Bayesian surprise, or Kullback–Leibler divergence, between the prior and posterior distribution over μ after a given trial. Let $\boldsymbol{x}$ denote the full sample of observed stimuli. The Bayesian surprise after the N-th trial is given by:

$$\begin{aligned}\text{surprise}_N^\tau\left(\boldsymbol{x}\right) &\equiv KL\left(f^\tau\left(\cdot|\boldsymbol{x}^N\right)|f^\tau\left(\mu|\boldsymbol{x}^{N-1}\right)\right)\\ &\equiv \int_0^1 f^\tau\left(\mu|\boldsymbol{x}^{N-1}\right)\log\frac{f^\tau\left(\cdot|\boldsymbol{x}^{N-1}\right)}{f^\tau\left(\mu|\boldsymbol{x}^N\right)}\mathrm{d}\mu.\end{aligned}$$

Due to the use of conjugate priors, the Kullback–Leibler divergence can be evaluated analytically, which significantly simplifies the computation. [1]

References

[1] Ostwald, D., Spitzer, B., Guggenmos, M., Schmidt, T. T., Kiebel, S. J., & Blankenburg, F. (2012). Evidence for neural encoding of Bayesian surprise in human somatosensation. *NeuroImage*, 62(1), 177–188. https://doi.org/10.1016/j.neuroimage.2012.04.050

[1] In our particular case, it can be shown that

$$\begin{aligned}\text{surprise}_N^\tau\left(\boldsymbol{x}\right) = &\log\frac{B\left(1+n_N^\tau, 1+m_N^\tau\right)}{B\left(1+n_{N-1}^\tau, 1+m_{N-1}^\tau\right)} + \left(n_N^\tau - n_{N-1}^\tau + m_N^\tau - m_{N-1}^\tau\right)\psi\left(2+n_{N-1}^\tau+m_{N-1}^\tau\right)\\ &- \left(n_N^\tau - n_{N-1}^\tau\right)\psi\left(1+n_{N-1}^\tau\right) - \left(m_N^\tau - m_{N-1}^\tau\right)\psi\left(1+m_{N-1}^\tau\right)\end{aligned}$$

where B is the Beta function introduced above and ψ is the Digamma function.
